# Development and psychometric validation of a home-based intravenous infusion needs scale and associated factors among Chinese older adults

**DOI:** 10.3389/fpubh.2026.1839972

**Published:** 2026-07-01

**Authors:** Mengmeng Yang, Yan Zhang, Yanan Zheng, Yue Li, Xueying Niu, Xuemei Li, Jinling Wu

**Affiliations:** 1 Cadre Medical Department, the First Medical Center of PLA General Hospital, Beijing, China; 2 Southern Medical Branch of PLA General Hospital, Beijing, China; 3 School of Nursing and Health, Henan University, Kaifeng, Henan, China

**Keywords:** associated factors, China, home-based intravenous infusion, needs assessment, older adults, psychometric validation, scale development

## Abstract

**Purpose:**

To develop and validate a scale for assessing home-based intravenous (IV) infusion needs among Chinese older adults, and to examine the level and determinants of such needs.

**Methods:**

The scale was developed in four stages: (1) item pool construction from the literature and semi-structured interviews with older adults; (2) initial scale development, including pilot testing and item revision; (3) scale validation, including expert evaluation of content validity, exploratory factor analysis, and reliability assessment in 303 older adults; and (4) application of the finalized scale to assess the level and determinants of home-based IV infusion needs.

**Results:**

The final scale comprised 19 items across four domains: professional and policy assurance, service delivery and quality assurance, safety and emergency assurance, and health guidance and follow-up assurance. These four factors accounted for 60.6% of the total variance. Item-level content validity indices ranged from 0.875 to 1.000, with scale-level indices of 0.895 (S-CVI/UA) and 0.987 (S-CVI/Ave). The total-scale Cronbach's α was 0.899. Item-level mean scores indicated a moderate-to-high level of need overall, with the highest scores observed for medical insurance reimbursement (4.12 ± 0.96) and healthcare providers' attitudes (4.10 ± 0.92). In multiple regression, living with spouse and children (vs. living alone; *B* = −0.290, β = −0.128, *P* = 0.030) and willingness to accept the service (*B* = 0.608, β = 0.357, *P* < 0.001) were independent predictors of need scores (adjusted *R*^2^ = 0.167).

**Conclusion:**

The scale showed good content validity, acceptable construct validity, and satisfactory internal consistency for assessing home-based IV infusion needs among Chinese older adults. Medical insurance reimbursement and healthcare providers' attitudes were the highest-rated needs. Living arrangement and willingness to receive home-based IV infusion were significantly associated with need scores. These findings may inform needs assessments, service planning, and reimbursement policies for home-based IV infusions. Further validation in larger and more diverse independent samples is needed.

## 1 Introduction

China accounts for approximately one-fifth of the global population aged 60 years and older (1). By 2030, a substantial share of this population—particularly rural residents and older women—is projected to experience severe functional dependency. Reduced mobility and diminishing family-based support will make hospital visits increasingly difficult for these individuals (2). Delivering medical services such as intravenous (IV) infusion in the home setting has therefore become an increasingly important issue. Nationally representative data from the China Research Center on Aging indicate that in-home physician visits rank as the most demanded community-based service among older adults, requested by 29.1% of respondents (3). This need appears particularly pronounced among older adults with chronic conditions. Wang et al. (4) surveyed 1,015 community-dwelling older adults in Beijing's Chaoyang District and found that 88.0% of those with chronic conditions reported a high need for home-based medical and nursing services. Routine nursing procedures, particularly IV infusion, were among the most commonly used services, and demand exceeded 35% among those with hypertension, hyperlipidemia, and diabetes. Polinski et al. (5) found that home-based IV infusion, beyond being safe and clinically effective, was associated with gains in quality of life and considerable cost savings.

In the U.S., where dedicated management systems and specialized nursing teams for home infusion are in place (6), the number of patients receiving home infusion therapy rose from 0.83 million in 2010 to over 3.2 million in 2019, representing a 310% increase (7). These services are also supported by established industry standards and regularly updated evidence-based clinical guidelines (8). In China, population aging has increased demand for home-based medical care (4). However, home-based IV infusion services in China remain insufficiently standardized, with substantial regional variation in patient selection, operating procedures, risk management, provider qualification requirements, and regulatory oversight (9). At present, home-based IV infusion in China is delivered mainly through two models: the home bed model and the “Internet + nursing services” model. The home bed model remains limited to pilot programs in coastal cities such as Shanghai and Shenzhen, with considerable variation across sites in infusion procedures, the types of medications considered suitable for home-based infusion, and quality oversight (10, 11). The “Internet + nursing services” model faces similar challenges, particularly the lack of unified industry guidance and operational standards, which may affect the consistency of safety management (12).

Against this background, an accurate assessment of older adults' needs for home-based IV infusion becomes particularly important. Such assessment is essential not only for identifying service priorities but also for informing the standardization and development of home-based IV infusion services. Existing geriatric needs assessment instruments, such as the Camberwell Assessment of Need for the Elderly (CANE), are mainly used to assess a broad range of health and social care needs among older adults (13). However, these instruments are not suitable for assessing needs related specifically to home-based intravenous (IV) infusion. In China, no validated, context-specific instrument is currently available to assess home-based IV infusion needs among older adults. This gap makes it difficult to assess such needs in a standardized manner and may limit the development of related services and policies (9, 14).

Against this background, this study had two objectives: to develop and validate a scale for assessing home-based IV infusion needs among Chinese older adults, and to apply the scale in a cross-sectional survey to examine the level and determinants of these needs.

## 2 Participants and methods

### 2.1 Study design

This study was guided by the scale development principles described by DeVellis et al. (15) and was organized into four stages. First, an item pool was constructed by synthesizing evidence from the literature with findings from semi-structured interviews with older adults. Second, the initial scale was developed through pilot testing and item revision. Third, the scale underwent psychometric evaluation, including expert assessment of content validity as well as exploratory factor analysis and internal consistency reliability testing in a sample of 303 older adults. Finally, the finalized scale was applied in a cross-sectional survey to assess the level and determinants of home-based IV infusion needs. The study was approved by the Institutional Review Board of Chinese PLA General Hospital (Approval No. S2021-134-01).

### 2.2 Item pool construction

#### 2.2.1 Literature review and initial framework

A targeted review of Chinese and international policy documents, clinical guidelines, and expert consensus statements related to home-based IV infusion was conducted (8, 10, 11, 16, 17). Content relevant to home-based IV infusion needs was extracted and synthesized to develop the initial framework.

#### 2.2.2 Semi-structured interviews

Older adults in Beijing were recruited using purposive sampling. Eligibility criteria included age 60 years or older, preserved cognitive function and adequate communication ability, written informed consent, and actual or anticipated need for home-based IV infusion services. Maximum variation sampling was used to capture diversity in age, gender, living arrangement, education, and occupation. Data collection and analysis proceeded concurrently. Saturation was considered reached when no new themes emerged in two consecutive interviews, and two additional interviews were then conducted to confirm saturation, resulting in a final sample of 14 participants. Participant characteristics are presented in Table 1.

**Table 1:** General information of interview participants (*n* = 14).

Participant ID	Age	Gender	Educational attainment	Former occupation
N1	62	Female	Junior high school	Farmer
N2	68	Male	High school	Manual worker
N3	75	Female	Bachelor's degree	Teacher
N4	65	Female	Primary school	Farmer
N5	65	Female	Secondary specialized school	Teacher
N6	64	Male	Bachelor's degree	Engineer
N7	62	Male	Associate degree	Teacher
N8	63	Female	Junior high school	Self-employed
N9	68	Female	Associate degree	Civil servant
N10	65	Male	Technical secondary school	Manual worker
N11	66	Female	Bachelor's degree	Physician
N12	67	Male	Associate degree	Engineer
N13	76	Female	Associate degree	Nurse
N14	88	Male	Bachelor's degree	Manual worker

Educational attainment was classified according to the Chinese education system. Primary school refers to 6 years of compulsory education; junior high school to 3 years of lower secondary education; high school to 3 years of upper secondary education; technical secondary school to vocational upper secondary education; associate degree to 3 years of post-secondary vocational education; and bachelor's degree to 4–5 years of undergraduate education. Participant IDs (N1–N14) were used to preserve anonymity.

The interview guide was developed on the basis of the literature review, the study objectives, and discussions within the research team. Pilot interviews with four older adults were conducted to refine the wording and sequence of the questions. The final interview guide consisted of nine core questions: (1) Are you aware of home-based intravenous infusion as a form of medical service? (2) Under what circumstances would you choose home-based intravenous infusion? (3) What factors do you think would influence your decision to choose home-based intravenous infusion? (4) What types of medications would you like to be included in the scope of home-based intravenous infusion services? (5) What needs do you have regarding the selection of infusion devices and equipment? (6) Apart from the intravenous infusion procedure itself, what specific services would you like medical and nursing staff to provide? (7) What expectations do you have regarding the quality of home-based intravenous infusion services? Please rank these expectations in order of importance. (8) How would you like the cost of home-based intravenous infusion to be determined? (9) What concerns or worries do you have about home-based intravenous infusion?

Before each interview, the researcher explained the study purpose, interview procedures, and confidentiality protections, and obtained consent for audio recording. A standardized explanation of home-based IV infusion was then provided, including its definition and common clinical indications. The interviews were conducted in a private setting and lasted 20–30 minutes. A reflective diary was completed after each interview. After the interviews, the audio recordings were transcribed into text and analyzed using content analysis in NVivo 14.

#### 2.2.3 Integration of literature review and interview findings

Findings from the literature review and semi-structured interviews were used to construct the initial item pool. Items with similar content were combined, and the wording of some items was standardized. Items identified in the interviews but not represented in the literature were retained. The resulting preliminary item pool was then used for pilot testing.

### 2.3 Initial scale development

The initial item pool was converted into a preliminary scale using a five-point Likert response format (1 = not at all needed to 5 = very much needed). A pilot study was then conducted with 50 older adults who met the same eligibility criteria to assess item clarity, comprehensibility, and response burden. Based on participant feedback and item analysis, items were revised and reduced before formal psychometric evaluation.

### 2.4 Psychometric evaluation

#### 2.4.1 Content validity

Content validity was assessed by a panel of eight experts, including three specialists in community nursing and five specialists in geriatric nursing. Four experts held senior professional titles and four held intermediate titles; five held graduate degrees and three held undergraduate degrees. Their ages ranged from 34 to 52 years, and their professional experience ranged from 11 to 30 years. Each expert rated the relevance of each item to the target construct on a four-point scale (1 = not relevant, 2 = slightly relevant, 3 = quite relevant, 4 = highly relevant). The Item-Level Content Validity Index (I-CVI) and the scale-level content validity index using both the universal agreement and averaging approaches (S-CVI/UA and S-CVI/Ave) were calculated (18).

#### 2.4.2 Participants and sample size

Between June 2025 and January 2026, older adults were recruited from Beijing, Hebei, and Hubei using convenience sampling. Questionnaires were completed either electronically or with researcher assistance, depending on participants' digital literacy.

Inclusion criteria. Participants were eligible if they: (1) were aged 60 years or older; (2) were able to understand and respond to the questionnaire, with or without assistance; (3) were cognitively intact and able to communicate effectively; (4) required home-based IV infusion services; and (5) provided informed consent. The exclusion criterion was withdrawal at any stage.

Sample size. Sample size determination was guided by methodological recommendations for exploratory factor analysis. A sample of 200 is generally considered adequate, whereas 300 or more is considered good (19). In addition, a participant-to-item ratio of at least 10:1 is commonly recommended (20). With 19 scale items, the minimum required sample was 190, and the target sample size was 300.

Data collection and exclusion criteria. A total of 332 questionnaires were collected. Questionnaires were excluded if: (a) completion time < 200 s; (b) identical responses for all items; (c) obvious logical inconsistencies; (d) missing any item. Excluded questionnaires were not imputed. This yielded 303 valid responses (valid rate 91.3%).

#### 2.4.3 Construct validity and reliability

Construct validity was examined using exploratory factor analysis (EFA). Before factor extraction, the suitability of the data for factor analysis was assessed using the Kaiser-Meyer-Olkin (KMO) measure and Bartlett's test of sphericity. Factors were extracted by principal component analysis with varimax rotation, retaining those with eigenvalues > 1. Items with factor loadings < 0.5 or substantial cross-loadings were considered for removal. Internal consistency reliability was assessed using Cronbach's α for the total scale and each dimension.

### 2.5 Scale finalization

The scale was finalized on the basis of the content validity, factor analysis, and reliability results. The finalized instrument was then used to assess the level of home-based IV infusion needs and their associated factors in the study sample.

### 2.6 Analysis of quantitative data

IBM SPSS version 27.0 was used for all analyses. Continuous variables are presented as mean ± standard deviation (SD). Normality was assessed by visual inspection of Q-Q plots and examination of skewness coefficients. For univariate comparisons of mean need scores, independent-samples t-tests were used for dichotomous variables, and one-way analysis of variance (ANOVA) was used for variables with three or more categories. Multiple linear regression was performed to examine factors associated with the mean need score, with all selected variables entered into the model simultaneously using the enter method. Given the exploratory nature of this study, variables with *P* < 0.10 in the univariate analyses were included in the multivariable model to reduce the risk of prematurely excluding potentially relevant factors (21). Multicollinearity was assessed using the variance inflation factor (VIF), with values < 5 considered acceptable.

## 3 Results

### 3.1 Item pool construction

The literature review identified three dimensions—professional and policy assurance, safety and emergency assurance, and health guidance and follow-up assurance—comprising 11 items. Content analysis of the 14 interviews identified four dimensions and 16 items, including an additional dimension, service delivery and quality assurance, that did not emerge from the literature. After integrating the items generated from the literature review and semi-structured interviews and removing overlapping items, a preliminary item pool comprising 20 items across four dimensions was constructed.

### 3.2 Initial scale development

Pilot testing of the provisional 20-item scale in 50 older adults indicated that the items were generally clear and acceptable. Based on participant feedback and item analysis, one item concerning reasonable and transparent fees was removed because it overlapped conceptually with an existing item addressing medical insurance reimbursement. The remaining 19 items were retained for subsequent psychometric evaluation. The contents of each item and their mean ± standard deviation values are presented in Table 2.

**Table 2:** Items of the home-based IV infusion needs scale (*n* = 19).

Item	Mean ±SD
1. Would you need healthcare professionals to conduct a comprehensive assessment of your health status, home environment, and suitability for home-based IV infusion before service delivery?	3.89 ± 0.96
2. Would you need healthcare professionals to explain basic IV infusion procedures (e.g., needle removal, sealing the infusion line, and post-removal compression) using easy-to-understand language or video demonstrations?	3.73 ± 0.98
3. Would you need home-based IV infusion services when you are physically frail, have limited mobility, or are unable to care for yourself?	4.07 ± 0.87
4. Would you need healthcare professionals to provide home-based IV infusion services promptly?	3.92 ± 0.87
5. Would you need home-based IV infusion services to be delivered by licensed healthcare professionals who are capable of independent practice and emergency response?	3.96 ± 0.93
6. Would you need healthcare professionals to provide care with a friendly and patient manner during service delivery?	4.10 ± 0.92
7. Would you need home-based IV infusion when the medications administered are intended for nutritional support or emergency treatment?	3.75 ± 1.11
8. Would you need healthcare professionals to plan venous access based on your medication regimen rationally?	3.80 ± 0.98
9. Would you need healthcare professionals to strictly adhere to operational protocols during the home-based IV infusion process?	4.03 ± 0.91
10. Would you need healthcare professionals to observe you for at least 30 minutes after the first administration of a medication and explain relevant precautions to you or your family members?	3.85 ± 1.01
11. Would you need healthcare professionals to closely monitor you for adverse reactions (e.g., allergies or fever) during infusion?	3.83 ± 1.11
12. Would you need timely access to professional medical assistance in case of emergencies during home-based infusion?	3.94 ± 1.06
13. Would you need healthcare professionals to safely dispose of sharps (e.g., needles and ampoules) and other hazardous medical waste generated during the infusion process?	3.55 ± 1.26
14. Would you need healthcare professionals to conduct regular follow-up visits after infusion to monitor treatment outcomes and recovery progress?	3.85 ± 0.94
15. Would you need precision infusion sets to minimize the introduction of air and impurities into the bloodstream?	3.84 ± 0.98
16. Would you need infusion pumps to control the infusion rate precisely?	3.57 ± 0.96
17. Would you need healthcare professionals to provide appropriate emergency equipment and medications based on your condition and the type of medication administered?	3.93 ± 0.95
18. Would you need the costs of home-based IV infusion to be covered by medical insurance reimbursement?	4.12 ± 0.96
19. Would you need home-based IV infusion services to be subject to national regulations ensuring standardized procedures?	3.94 ± 0.95

### 3.3 Scale validation

#### 3.3.1 Content validity

The item-level content validity index (I-CVI) values ranged from 0.875 to 1.000, all exceeding the established threshold of 0.78 for acceptable item-level validity (22). At the scale level, the scale-level content validity index based on the universal agreement approach (S-CVI/UA) was 0.895, surpassing the recommended criterion of ≥0.80 (23). The scale-level content validity index based on the averaging approach (S-CVI/Ave) was 0.987, substantially exceeding the recommended standard of ≥0.90 (24). Taken together, these findings support the content validity of the questionnaire at both the item and scale levels.

#### 3.3.2 Construct validity and reliability

Internal consistency reliability was satisfactory. Cronbach's α was 0.899 for the total scale, and the coefficients for the four dimensions ranged from 0.705 to 0.863. Corrected item-total correlations ranged from 0.417 to 0.643, with all items exceeding the commonly recommended threshold of 0.30 (15). Inter-item correlations ranged from 0.125 to 0.638. Most item pairs (154/171, 90.1%) fell within the recommended range of 0.15–0.50, indicating adequate homogeneity without substantial redundancy (25). Although a small number of item pairs fell outside this range, the corrected item-total correlations, internal consistency coefficients, and item-deletion results did not indicate a need for further item removal.

The Kaiser-Meyer-Olkin value was 0.909, and Bartlett's test of sphericity was significant (χ^2^ = 2366.781, df = 171, *P* < 0.001), supporting the suitability of the data for factor analysis. Principal component analysis with varimax rotation extracted four factors with eigenvalues greater than 1, which together explained 60.6% of the total variance. All items loaded above 0.50 on their intended factors, and no cross-loadings were observed. The factor loading matrix is presented in Table 3.

**Table 3:** Rotated factor loadings of items in the home-based IV infusion needs sale.

Item	Safety and emergency assurance	Service delivery and quality assurance	Professional and policy assurance	Health guidance and follow-up assurance	Cronbach's α for each dimension
Item 17	0.744				0.829
Item 12	0.722				
Item 10	0.683				
Item 15	0.663				
Item 11	0.644				
Item 13	0.618				
Item 16	0.615				
Item 6		0.772			0.863
Item 3		0.763			
Item 8		0.761			
Item 4		0.756			
Item 9		0.678			
Item 19			0.832		0.828
Item 18			0.804		
Item 5			0.648		
Item 1			0.625		
Item 2				0.705	0.751
Item 7					0.733
Item 14					0.716

Only factor loadings ≥0.50 are shown. Factors were extracted using principal component analysis with varimax rotation. Dimension α indicates Cronbach's α for each dimension.

### 3.4 Scale finalization and cross-sectional application

The final scale comprised 19 items across four dimensions: professional and policy assurance, service delivery and quality assurance, safety and emergency assurance, and health guidance and follow-up assurance. It was then used to assess the level of home-based IV infusion needs and their associated factors in the sample of 303 older adults.

#### 3.4.1 Univariate analysis

Univariate analysis was performed with the mean need score as the dependent variable. Significant differences were observed for living arrangement and perceived need for home-based IV infusion (*P* < 0.05). No statistically significant associations were found for gender, age, educational attainment, Former occupation, marital status, presence of chronic diseases, health status, mobility, place of residence, awareness of home-based IV infusion, or monthly income (*P* > 0.05). Descriptive characteristics and univariate analysis results are presented in Table 4.

**Table 4:** Univariate analysis of factors associated with home-based IV infusion needs.

Item	Category	*n*	Mean ±SD	t/F	*P*
Gender	Male	120	3.86 ± 0.54	−0.402	0.688
	Female	183	3.89 ± 0.62		
Age	60-69	227	3.87 ± 0.588	−0.462	0.644
	≥70	76	3.90 ± 0.63		
Educational attainment	Primary school or below	47	3.99 ± 0.085	1.004	0.406
	Junior high school	100	3.90 ± 0.06		
	High school/technical secondary school/vocational high school	85	3.80 ± 0.07		
	Associate degree	43	3.83 ± 0.06		
	Bachelor's degree or above	28	3.95 ± 0.08		
Former occupation	Manual laborer	148	3.94 ± 0.57	1.425	0.242
	Enterprise/public institution employee	76	3.81 ± 0.54		
	Unemployed/self-employed	79	3.83 ± 0.66		
Marital status	Unmarried/widowed/divorced	35	3.76 ± 0.78	−0.975	0.336
	Married	268	3.89 ± 0.56		
Health status	Good	197	3.91 ± 0.58	2.739	0.066
	Fair	86	3.77 ± 0.62		
	Poor	20	4.06 ± 0.45		
Chronic disease status	No	174	3.93 ± 0.50	1.743	0.082
	Yes	129	3.81 ± 0.69		
Mobility	Independent	253	3.86 ± 0.61	−1.308	0.192
	Needs assistance	50	3.98 ± 0.44		
Residence	Urban	169	3.86 ± 0.61	−0.653	0.515
	Rural/town	134	3.90 ± 0.56		
Living arrangement	Living alone	74	4.02 ± 0.48	3.575	**0.014**
	Living with spouse only	162	3.88 ± 0.57		
	Living with children only	45	3.76 ± 0.64		
	Living with spouse and children	22	3.61 ± 0.84		
Monthly income	≤ 2,000 RMB	75	3.89 ± 0.62	0.841	0.432
	2,001–5,000 RMB	152	3.91 ± 0.60		
	≥5,001 RMB	76	3.80 ± 0.52		
Awareness of home-based IV Infusion	Not aware	170	3.82 ± 0.59	−1.962	0.051
	Aware	133	3.95 ± 0.58		
Willingness to receive home-based IV infusion	Unwilling	42	3.33 ± 0.90	−7.042	**< 0.001**
	Willing	261	3.97 ± 0.47		

A two-tailed P-value < 0.05 was considered statistically significant in the univariate analysis. Bold values indicate statistical significance. SD, standard deviation; t, t-test statistic; F, F-test statistic; P, p-value.

#### 3.4.2 Multivariate regression

Multiple linear regression (enter method) was performed to identify independent predictors of home-based IV infusion needs. The dependent variable was the mean need score. Variables with *P* < 0.10 in univariate analysis (health status, chronic disease status, living arrangement, awareness of home-based IV infusion, and perceived need for home-based IV infusion) were entered as independent variables. Unordered categorical variables were dummy-coded in Table 5.

**Table 5:** Dummy coding of categorical variables included in the regression model.

Independent variable	Coding
Health status	Good = 000, Fair = 010, Poor = 001
Living arrangement	Living alone = 0000, Living with souse only = 0100,
	Living with children only = 0010, Living with spouse and children = 0001

Dummy coding was applied to the categorical variables included in the regression model. Good health status and living alone served as the reference categories and were coded as all zeros.

The multivariable linear regression model was statistically significant (*F* = 8.571, *P* < 0.001) and yielded an adjusted *R*^2^ of 0.167, indicating that the included variables explained 16.7% of the variance in mean need scores. Compared with living alone, living with a spouse and children was associated with lower need scores (*B* = −0.290, β = −0.128, *P* = 0.030), whereas willingness to accept home-based IV infusion services was associated with higher need scores (*B* = 0.608, β = 0.357, *P* < 0.001). Variance inflation factors ranged from 1.014 to 1.514, suggesting no evidence of multicollinearity. Residual diagnostics indicated approximate normality of the residuals, and the Durbin-Watson statistic of 1.585 suggested no substantial autocorrelation. Detailed results are presented in Table 6.

**Table 6:** Multivariate analysis of factors associated with home-based IV infusion needs among older adults.

Variable	B	Beta	*t*	*P*	95.0% Confidence Interval		VIF
Constant	2.807		12.157	< 0.001	2.353	3.261	–
Health status (reference: good)							
Fair	−0.117	−0.090	−1.625	0.105	−0.259	0.025	1.104
Poor	0.144	0.061	1.095	0.274	−0.115	0.403	1.120
Living arrangement (reference: living alone)							
Living with spouse only	−0.132	−0.112	−1.735	0.084	−0.282	0.018	1.514
Living with children only	−0.195	−0.118	−1.893	0.059	−0.397	0.008	1.403
Living with spouse and children	−0.290	−0.128	−2.175	**0.030**	−0.552	−0.028	1.250
Chronic disease status (reference: no)	−0.048	−0.041	−0.727	0.468	−0.179	0.083	1.136
Awareness of home-based IV infusion (reference: not aware)	0.106	0.090	1.694	0.091	−0.017	0.230	1.014
Willingness to receive home-based IV infusion (reference: unwilling)	0.608	0.357	6.661	**< 0.001**	0.428	0.787	1.041
*R*^2^ = 0.189 adjusted *R*^2^=0.167	–	–	–	–	–	–	–
*F* = 8.571	–	–	–	–	–	–	–
*P* < 0.001	–	–	–	–	–	–	–

Dependent variable: mean score for home-based IV infusion needs. Bold values indicate p < 0.05. B, unstandardized coefficient; β, standardized coefficient; t, t statistic; p, p-value; VIF, variance inflation factor; R^2^, coefficient of determination; adjusted R^2^, adjusted coefficient of determination; F, F statistic; CI, confidence interval.

## 4 Discussion

### 4.1 Development rigor and psychometric evidence

The present scale is the first instrument specifically developed to assess home-based IV infusion needs among Chinese older adults. Its development followed a four-stage process: item pool construction, initial scale development, psychometric evaluation, and finalization (26). The item pool was informed by both published evidence and semi-structured interviews with older adults. The literature review, drawing primarily on clinical guidelines and policy documents, provided an initial framework encompassing professional and policy assurance, safety and emergency assurance, and health guidance and follow-up assurance. While the interview findings supported these domains, they also identified service delivery and quality assurance as a distinct dimension that had not been separately represented in the literature-based framework. This additional dimension suggests that older adults' needs for home-based IV infusion extend beyond technical and safety considerations to include expectations regarding service delivery and quality of care.

The scale demonstrated good psychometric properties. The four-factor structure identified by exploratory factor analysis explained 60.6% of the total variance (27) and was broadly consistent with the theoretical framework developed during scale construction, providing preliminary support for the multidimensional structure of home-based IV infusion needs among Chinese older adults. The total scale and all four dimensions also showed satisfactory internal consistency reliability, with a Cronbach's alpha of 0.899 for the overall scale and coefficients ranging from 0.705 to 0.863 across dimensions. Although the Cronbach's alpha coefficient for the health guidance and follow-up assurance dimension was relatively lower, this may be related to the small number of items in that dimension (28). In addition, the high content validity indices indicated good expert agreement regarding the relevance and representativeness of the items (22–24). Overall, these findings support the use of the scale as a preliminary instrument for assessing home-based IV infusion needs among Chinese older adults.

These findings are broadly consistent with those reported for other needs assessment instruments developed for older adult populations in China. For example, Wang et al. (29) reported a total-scale Cronbach's α of 0.832, an I-CVI of 0.83–1.00, and an S-CVI of 0.982 for a home hospice care needs questionnaire. Although that instrument addressed a different care context, the similarity in reliability and content validity indices suggests that the present scale performs at an acceptable level while focusing on a more procedure-specific domain. Further work is still needed to refine the health guidance and follow-up assurance dimension and to examine the stability of the factor structure in more diverse samples.

### 4.2 Scale structure and comparison with existing instruments

The four dimensions of the scale reflect the main stages of home-based IV infusion care, including professional and policy assurance before service initiation, service delivery and quality assurance during care provision, safety and emergency assurance throughout the infusion process, and health guidance and follow-up assurance after infusion. Among them, safety and emergency assurance was the largest dimension, comprising seven of the 19 items. Items in this dimension, such as adverse reaction monitoring, emergency equipment availability, safe sharps disposal, and infusion pump precision, correspond closely to the core safety components of home infusion practice identified by Gorski (30), including patient monitoring, emergency preparedness, infection prevention, and safe use of infusion equipment. This correspondence supports the clinical relevance of the dimension. The prominence of safety-related items also reflects a defining feature of home-based IV infusion. When an invasive procedure is delivered at home rather than in a hospital, the management of clinical risk relies more heavily on patients, family members, and visiting providers, who work without the full support of an institutional setting (31).

Existing needs assessment instruments, both international and Chinese, have generally been designed to capture broader health, social care, or home care needs rather than the requirements of a specific clinical procedure. CANE, for example, is a validated screening tool for general health and social care needs among older adults (32, 33). It does not address issues central to home-based IV infusion, such as emergency management of infusion-related adverse events, safe sharps disposal, infusion equipment precision, or reimbursement arrangements that influence service access. Similarly, two instruments developed in the Chinese context—an assistance needs scale for community-dwelling older adults (34) and a social care needs tool for end-stage patients receiving home-based care (35)—focus on broader home-based care needs rather than the demands associated with a specific medical procedure. The present scale provides a structured way to assess needs specific to home-based IV infusion. Its four-domain framework may also inform the development of procedure-specific assessment tools for other home-based services. In practice, it may help nurses and service providers identify areas of greatest need among older adults and support more focused care planning.

### 4.3 Key findings and implications

In this cross-sectional survey, medical insurance reimbursement and providers' attitudes were the two highest-rated aspects of home-based IV infusion needs. Medical insurance reimbursement received the highest mean score (4.12 ± 0.96), which may reflect the economic profile of the study sample, as shown in Table 4, where 74.9% of participants reported a monthly income below 5,000 RMB. This finding suggests that affordability is a key consideration in shaping perceived need for home-based IV infusion services. Providers' interpersonal attitudes ranked second (4.10 ± 0.92), indicating that older adults value not only financial accessibility but also the quality of interpersonal care. Together, these findings highlight that both economic protection and person-centered care delivery are important for promoting acceptance of home-based IV infusion services (36).

Living situation and willingness to accept services each independently predicted need scores. Need was highest among older adults living alone and lowest among those living with both spouse and children (37, 38). Zeng et al. (39) reported the same pattern: in a national sample of Chinese older adults, those who lived alone expressed stronger demand for home- and community-based services than those living with family, across physical, psychological, and social domains alike. Living alone means fewer caregivers available for daily health tasks and greater worry about a medical emergency with no one present — both of which would raise perceived need for formal home-based services.

Willingness to receive home-based IV infusion services was the strongest predictor in the regression model (β = 0.357). Mean need scores were markedly higher among participants willing to receive the service than among those unwilling to do so (3.97 ± 0.47 vs. 3.33 ± 0.90). This finding has an important practical implication: needs assessment strategies that do not take willingness into account may misclassify actual service demand. Service planners should distinguish between older adults who need the service and those who are willing to use it, as these two groups do not fully overlap (40). Incorporating willingness screening into routine assessment may help direct resources toward individuals with both objective need and subjective readiness. The regression model explained only 16.7% of the variance in home-based IV infusion needs, indicating that additional determinants remain to be identified. Future studies should examine factors such as prior experience with home-based services, trust in community providers, and local service availability.

## 5 Conclusion

This study developed and validated a 19-item, four-dimensional scale for assessing home-based IV infusion needs among Chinese older adults. Psychometric evaluation supported the instrument's reliability and validity. The cross-sectional application identified financial protection and humanistic care as priority concerns and showed that living arrangements and willingness to accept services independently predict need. These findings offer an empirical basis for service delivery planning and reimbursement policy design. Further multicenter validation in geographically diverse samples is needed to strengthen generalizability.

### 5.1 Limitations

Limitations should be acknowledged. First, convenience sampling across three provinces (Beijing, Hebei, and Hubei), together with the recruitment of all interview participants from Beijing, may limit the geographic representativeness of both the item pool and the survey findings; therefore, the needs of older adults in western or more remote regions may not have been fully captured. Second, the cross-sectional design precludes causal inference, and the psychometric evaluation was limited to exploratory factor analysis and internal consistency reliability; confirmatory factor analysis in an independent sample and test-retest assessment are still needed further to verify the scale's structural stability and temporal reliability. Third, content validity was assessed through a single round of expert ratings, and additional refinement through a multi-round Delphi process may further strengthen item wording and content coverage. Finally, the regression model explained only 16.7% of the variance in home-based IV infusion needs, suggesting that other unmeasured factors may also contribute to this demand. Future studies should validate the scale in larger, more geographically diverse samples, incorporate confirmatory factor analysis and test-retest reliability assessments, and further refine items using Delphi methods.

## Data Availability

The original contributions presented in the study are included in the article, further inquiries can be directed to the corresponding author.
